# Mr. Burns' Surgical Anatomy of the Head and Neck

**Published:** 1816-04

**Authors:** 


### *Mr. Burns' Surgical Anatomy of the Head and Neck.*

( Continued from page 250. )

Tracheotomy.—This operation may be required either to admit air into the lungs, or for the removal of a foreign body from the trachea: and, on reverting to the peculiarity of structure which distinguishes the throat of the young subject, we shall perceive that it may be performed with equal facility at that, as at the adult age. It was formerly much recommended in the different species of asphyxia; but the expert surgeon will seldom have occasion to employ it in these cases. The introduction of a curved tube along the floor of the *right* nostril into the opening of the glottis, as proposed by Desault, is far preferable, and readily effected by those who have an accurate knowledge of the structure and relations of the parts about the throat. In this way, the obstacle opposed by the epiglottis to the passage of the instrument through the mouth, will be avoided; for the point of the tube passes down " behind the line of that valve." The introduction will be accomplished with greater facility, if the tongue be drawn forward, and the *basis cranii* be placed parallel to the horizon.* With respect to the best situation for making an opening into the wind-pipe, dif-

---

* In passing the probang, the tongue should be permitted to fall back into the mouth; for thus the epiglottis will be brought behind the line of the *velum*, and the instrument, striking the valve, will fold it over the glottis. The same remark will apply to the introduction of a tube by the nostril into the gullet; but, in the former case, the head should be turned back; in the latter, be kept parallel with the horizon.

ferent opinions have existed. Some have preferred the larynx; others, that part of the trachea below the thyroid gland. The point most commonly selected for *laryngotomy* is the interval between the thyroid and cricoid cartilages. We have seen it performed in the centre of the former of these cartilages, but certainly without any advantage that could compensate for the difficulty of penetrating this hard and frequently ossified body, or for the danger, obviously incurred, of wounding the *chordæ vocales*. Perforation of the larynx between the cartilages is considered objectionable by Mr. Burns, as being too near the *rima glottidis*; and hence likely to excite great irritation and coughing. Tracheotomy is preferable, especially in young subjects, and women, with whom, as we have before remarked, the developement of the larynx bears no proportion to that displayed by the vocal organ of the adult male. Yet tracheotomy has its difficulties: for not only may the great subclavian vein, and the inferior thyroideal vessels, *arteria innominata*, or carotids, in consequence of slight deviation from their wonted course, be endangered,— but large vessels, of irregular origin and distribution, may be directly exposed,—in this operation. Mr. Burns cites several examples of this nature. He once saw the cross slip, which connects the lobes of the thyroid gland in front of the trachea, so broad as to descend "almost to the sternum." These circumstances should be recollected by the surgeon in operating on the trachea. They will remind him to employ the knife in the division of the integuments and fascia only, and to use his finger in clearing the trachea, and in depressing toward the chest any large vessels that may be in his way, ere he perforate the tube. The incision of the trachea should be made in the longitudinal direction, and from below upward, with care to guard from injury the thyroid gland.

*Aneurism.*—The observations of Mr. Burns on this subject may be arranged under two heads:—1. History of the operation for aneurism and general doctrines respecting it. 2. Particular remarks and cases; and this second division will admit of being farther distributed into three sections, under the respective titles of Operation on the subclavian artery; on the carotid artery; and aneurism from anastomosis. We, however, deem it proper to apprize the reader, that this is our own arrangement, of which he will find nothing but the detached and often widely-scattered materials in the work of Mr. Burns. Upon the history and general doctrines of aneurism, though discussed at considerable length by Mr. Burns, we think it unnecessary to enter very diffusely, after the minute and comprehensive manner in which the luminous observations of Mr. Hodgson on this important topic have recently been reviewed by us :* more especially, as the labors of an enlightened predecessor, such as Mr. Burns, would be, and evidently have been, turned to account by the London pathologist: and it is, moreover, a subject to which we shall presently revert in concluding our Analysis of the Medical Sketches of Mr. Cross. Under these circumstances, it may suffice to trace a rapid outline of Mr. Burns' opinions on aneurism ; and note those leading points of doctrine in which he coincides with, or differs from, other distinguished writers. We shall present them under the few following heads. First, Mr. Burns believed that Dr. Jones was mistaken in considering the division of the two internal coats of an artery *essential* to the obliteration of the vessel. In this opinion we coincide with Mr. Burns. Were it otherwise, on what principle are we to explain the occurrence of spontaneous obliteration, frequently observed ; or the successful issue of the operations for aneurism by Scarpa, Assalini, Crampton,† and the French surgeons, where no division of the arterial coats could have taken place? Secondly, He considers employment of the double ligature, and division of the vessel in the interspace, as recommended by Mr. Abernethy, advisable: and that irritation will be diminished by removing one end of each ligature, and cutting out the portion of artery intercepted between the ligatures, if a greater length than necessary have been insulated in the operation. Thirdly, All topical stimulants, applied with the view of supporting the temperature of a limb, after the operation for aneurism, and while the energies of circulation remain languid, are pernicious. That the limb, during this period of local debility, may suffer, and even fall into gangrene, from the administration of *internal* stimuli, is proved by a case which Mr. Burns had witnessed. A member, thus circumstanced, is nearly in the situation of an organ "benumbed with cold :" And we are not only to guard against the ill consequences of "over-exciting the limb, but we

* See "New Med. and Phys. Journal," vol. x. page 488.

† See New Med. and Phys. Journal, vol. ix. page 512; and vol. x. page 510.

must be watchful to keep the action of the system in general, moderate." These observations are, on the whole, very judicious. Fourthly, We cannot speak so favorably of his strictures on the proposal of Desault to pass a ligature round an aneurismal artery at the *remote* extremity of the sac, in those cases where, from the unfavorable situation of the aneurism, the portion of vessel between it and the heart is inaccessible. This operation is mentioned by Mr. Burns in terms of utter contempt and reprobation; as calculated only to produce a more rapid enlargement of the tumor; in fact, as "absurd in theory, and ruinous in execution." We are, ourselves, as fully aware as Mr. Burns could have been, of the precarious nature of such an operation; since, in addition to the ordinary sources of failure of the ligature in the more common situations of aneurism, the accidental origin of an arterial branch from the sac itself, or from the vessel, between the ligature and the sac, would here completely foil the efforts of the operator. Yet be it remembered, that this is an operation proposed only in cases otherwise completely desperate and forlorn,—in cases where nothing but destruction, more terrible from its suddeness and uncertainty, awaits the devoted patient. Under circumstances thus deplorable, any attempt, however faint be the prospect of relief which it offers, is at least warrantable. And it is surely unjust and unphilosophical wholly to proscribe this operation on account of failure in the few instances wherein it has yet been tried. Where would have been the pride and boast of British surgery in the treatment of aneurism, had the disastrous termination of Mr. Abernethy's early attempts upon the carotid and external iliac arteries been regarded as decisively fixing a fatal character upon all such "absurd" and "ruinous" experiments? Indeed, we agree with Mr. Hodgson, that "the termination of the two cases in which this practice has been tried, by no means destroys the probability of a happier result under other" (more favorable) "circumstances;"* nor would we hesitate to propose it in the first case of subclavian or external iliac aneurism that may fall within the sphere of our observation. Lastly, the views of Mr. Burns respecting the application and efficacy of compression in the treatment of aneurism, and its mode of operation, are scarcely so clear and correct

* See Hodgson's Treatise on the Diseases of Arteries, &c. page 302.

as those of Mr. Hodgson ; and we think, that he did not adequately appreciate the merits of Valsalva's lowering plan in the cure of this formidable malady, or lay sufficient stress upon its employment. The particular remarks of Mr. Burns, as they regard two different situations of common aneurism ; and a peculiar form of lesion possessing some of the characters of aneurism, now remain to be examined. We shall adopt the order wherein the sections, under which we have arranged them, were before announced.

*Operation on the Subclavian Artery.*—To any one who contemplates the connections of the subclavian artery, in the earlier part of its course, with important nerves* (on the left side, with the termination of the thoracic duct) and its contiguity to the pleura ; the difficulty and dangers of passing a ligature round the vessel on the tracheal side of the scaleni muscles are sufficiently obvious : and the operation assumes a yet more formidable character from the possibility of mistaking aortic for subclavian aneurism. A case is recorded with great candour and clearness, by Mr. Burns, in which this mistake was absolutely committed by many eminent and experienced surgeons. The tumor rose above the sternum in such a way, that no one entertained a doubt as to its having sprung from the right subclavian artery ; but, fortunately, the professional men were deterred from operating by uncertainty respecting the state of the *arteria innominata*. On the death of the patient, the aneurism was found to have arisen from the aorta. The *arteria innominata* was considerably implicated in it ; the subclavian but slightly dilated at its root ; the descending *vena cava*, thrust by it to the right ; the trachea and œsophagus, to the left ; and the roots of the left carotid and subclavian arteries compressed against the spine. The history altogether is most valuable and instructive, and is illustrated by an anterior and posterior view of this singular aneurism. The outline of a case is also given, in which a flask-like tumor, arising from the arch of the aorta between the left subclavian and carotid, had been mistaken for aneurism of the latter vessel. This fact came under the observation of Mr. Astley Cooper, from whom Mr. Burns first received the intimation respecting the possibility of confounding aortic aneurism with aneurism of the arteries of the neck.

---

* Eighth, recurrent laryngeal, sympathetic, and phrenic.

Of the ligature of the *arteria innominata,* considered practicable by Mr. Burns, little needs be said: for, although almost free from apprehension as to the consequences of intercepting the cerebral supply of blood by two channels at once, we can hardly conceive circumstances demanding such an operation. As an example of the possibility of passing a ligature round the subclavian artery directly after its passage from between the *scaleni* muscles, the case, operated upon by the late Mr. Ramsden, is quoted at length from his work.*

*Operation on the carotid artery.*—Carotid aneurism not unfrequently occurs; but is no longer the hopeles malady which, within a few years, it was considered. It commonly takes place at the point where the trunk of the vessel bifurcates, and often arises from a previous lesion of the arterial coats. Sometimes there is simple dilatation, not dependent on morbid structure. In cutting down upon the carotid above the point of decussation of the omo-hyoid and sterno-mastoid muscles, the vessel lies superficial: the integuments, platysma myoides, and fascia, are only to be divided. But, from the usual situation and size of carotid aneurism, the surgeon will be, in most cases, constrained to operate nearer the chest; where the artery is more deeply placed, and intimately connected with important parts. Here, after dividing the integuments, platysma myoides, and fascia along the anterior margin of the sterno-mastoid muscle, he will have to dissect back its sternal attachment, and turn it outward till the acromial border of the sterno-thyroideus comes into view. In raising this last muscle, some filaments of the *nervus descendens noni* will be divided. The sterno-mastoid and omo-hyoid muscles being drawn aside towards the acromion, and the sterno-hyoid and thyroid towards the trachea, by means of blunt hooks, the common sheath of the great cervical vessels and eighth nerve will be exposed, with the *descendens noni* lying upon its fore-part. The œsophagus also will be seen (if it be the left side) on the tracheal margin of the sheath, covered by twigs of the recurrent nerve. In opening the sheath, the trunk of the *descendens noni* should be avoided. The separation of the jugular vein from the artery will require considerable caution and address. The eighth nerve, from its size and density, is readily distinguished and detached; the sympathetic is secured by its close connection to the

* Practical Observations on Sclerocele, &c. page 276.

spine; but, where the operation is performed low down, on the left side, the termination of the thoracic duct will be in danger: for here it lies just behind the carotid, interposed between its sheath and the sympathetic nerve; and "in some subjects, it mounts pretty high in the neck before it curves downward and outward, to join the subclavian vein." An injury of this little vessel would be obviously irreparable. The *nervus superficialis cordis* is also to be respected. Mr. Burns recommends, that two ligatures should be put round the artery; that, under common circumstances, division of the vessel between them is an object of indifference; but that, if much of it have been insulated, the intermediate portion should be cut out. On this subject, however, we beg leave to quote a passage from Mr. Hodgson's work, as being perfectly in unison with our own opinions.

"The propriety of dividing the vessel in the interspace when the *carotid* artery is the subject of the operation, is still doubtful; for if the operation has been performed low in the neck, and the ligature should from any cause slip off, it will be impossible again to secure the vessel, or permanently to command the hæmorrhage."*

The surgeon, in operating upon the carotid, should also recollect that the vertebral artery has been known to accompany the former nearly to its bifurcation, and in contact with its sheath. Now, although, as before observed, we by no means feel the apprehensions expressed by Mr. Burns with respect to the consequences which might result from obstructing the passage of the blood to the brain by two of its channels,† it would obviously be rash and unwise to include the vertebral artery, without any object: and both this vessel, the thoracic duct, and all its important nerves exterior to the sheath, may to a certainty be avoided, if care be taken to pass the ligature *between* the carotid artery and the sheath. Another reason for remembering the anomaly of the vertebral artery is, that, were the vessel, thus irregularly situated, to become aneurismal, the tumor would assume all the delusive characters of carotid aneurism; and the surgeon scarcely discover his mistake until he had passed a ligature round the carotid. Hence, when the sheath of the vessels has been exposed,

---

* Hodgson's Treatise, Page 224.

† Both carotid arteries have been tied, at the same time, in the inferior animals, without evident injury of the functions of the brain.

we ought invariably to try what influence compression of the artery between the finger and thumb exercises upon the aneurismal tumor: if the pulsation of it be not in this way considerably diminished, great caution to avoid an error will become necessary. Again, the common carotid sometimes divides very low in the neck (even opposite to the sixth cervical vertebra). In this case, it is evident that, were the internal or external carotid to become the seat of aneurism, there could be no necessity for taking up both, or tying the common trunk, unless the disease were situated so low down as to require it. Mr. Burns concludes by transcribing at full length, from the first volume of the Medico-Chirurgical Transactions, Mr. Astley Cooper's successful case of ligature of the carotid artery.

*Aneurism from anastomosis* may be divided into two species: the one, in which the arteries are chiefly affected, an acute and perilous disease; the other, implicating the veins, chronic and less formidable. The former may originate from a congenital discoloured spot; from external violence, or without evident cause: it is, at first, small but gradually increases: the pulsation, once obscure, becomes stronger; sense of pain and distension comes on. At length, the whole tumor pulsates distinctly, and, when excited by muscular exertion, vehemently. It assumes a purple colour; the apices of the sacculi become thin; and the patient sinks exhausted by repeated hæmorrhage. A case is related to illustrate this species; and to shew, in opposition to the doctrine of Mr. John Bell, that *partial* excision of anastomosing aneurism may terminate successfully. It is essential, however, that the disease be seated over a bone, in order that permanent pressure may be employed. The aneurism, in the example given by Mr. Burns, was connected with the temporal artery, and extended deep beneath the zygoma. The sponge and twisted bandage were used as agents of compression. We suspect that, in tracing the origin of this affection, Mr. Burns has been betrayed into some very unsound reasoning. Unwilling to pause here so long as a full investigation of the subject would require, we shall, shortly, revert to it, in communicating a case, analogous in its nature, though not in the situation which the disease occupied. No separate history of the progress of venous anastomosing aneurism is delivered; but we have an instance detailed in which Mr. Burns successfully removed a tumor of this description, seated on the cheek, and implicating the *conjunctiva* of the eye, the temporal muscle, transvere artery of the face, principal branch of the facial nerve, and the parotid duct. Much diversity of opinion has existed respecting the structure of anastomosing aneurism; some contending that it is wholly composed of a congeries of convoluted vessels; others, among whom we may particularize Mr. Freer, appealing to direct experiment in proof of their assertion that it consists entirely of cells, like the placenta.* Mr. Burns, from his own observation, was "inclined to believe" that its structure was "really cellular." But the fallacy of his opinion, that "any attempt to cure this disease by ligature of the arteries which support it is quite out of the question," has since been most incontrovertibly proved by the splendid operation of Mr. Travers.

*Pathology of the tongue, tonsil, and saliv.ry glands.*—Ligature may be safely employed to remove portions of *the tongue*; yet sometimes the morbid parts are so situated as to render this operation impracticable. Under such circumstances, the trunks of the lingual arteries having been secured previously to their giving off any large branches,† extirpation with the knife might be safely resorted to. Ligature, however, if applicable, is certainly to be preferred. In dividing the frenum of children, "an operation oftener performed than needed," the points of the scissars should be turned downward, not upward and backward, in order to avoid the ranial artery lying "just above the attachment of the frenum."——*The tonsil*, from its vascularity, is exceedingly prone to disease, and often inflames and suppurates. The swelling, when large, obstructs respiration and deglutition by its mechanical pressure: hence vigorous measures should be employed to procure resolution; or, if these fail, to expedite the process of suppuration. The abscess commonly bursts between the pillars of the fauces, sometimes through the *velum palati*; and the sore which remains may be mistaken by an incautious observer for syphilis. When the abscess presents on the *velum*, the matter is deep-seated, and the patient may perish from suffocation, ere it be spontaneously discharged. As soon, therefore, as circumstances demand it, and obscure fluctuation be felt, the abscess should be punctured, with due precaution to

---

* Mr. John Bell asserts, that an artery opens into each cell, and a vein originates from it.

† See the Note at page 60, of our former Article.

push the instrument *into the front of the cyst and carry it directly backward instead of inclining it towards the angle of the jaw,* whereby the carotid artery, closely connected with the gland, might be penetrated. Instances of this blunder, followed by fatal hæmorrhage, have in reality occurred. By suffering the abscess to attain a very large size, patients have absolutely been destroyed, on its spontaneous rupture, by the deluge of purulent matter getting into the trachea. To prevent the possibility of such an occurrence in puncturing the tonsil, the operation may be performed by means of a trochar and canula. When, after repeated suppuration, the gland becomes indurated and so much enlarged as to produce unpleasant consequences; if topical bleeding, blisters, electricity, and the daily employment of a purgative salt fail to diminish it, the diseased mass must be removed. This may be accomplished by the knife or ligature. In the former operation, which is preferable, it is rarely necessary, nor would it be safe, to remove the whole tonsil: the hæmorrhage may be checked by the application of cold water, turpentine, alcohol, or, in urgent cases, by the actual cautery. The latter agent will also destroy the fungus which occasionally prevents cicatrization; if the employment of powdered muriate of ammonia have been tried in vain. Chronic enlargement of the gland sometimes depends on the formation of calculi: they are seldom large; but produce irritation, and repeated inflammatory attacks commonly ending in suppuration. When the active stage is past, a solid and circumscribed swelling remains; and the concretion may sometimes be detected by a probe. Three cases of this kind, occurring in individuals nearly related to each other, are mentioned. No attempt to extract the calculus by an external incision is warrantable, unless it have made its way directly under the skin. The substance, however, may be removed by dividing the gland internally, as soon as its existence is clearly known. Sometimes, the concretion is not solid; but may be "turned out from the recesses of the tonsil in gritty masses of a dirty white colour." The formation of this matter seems to be connected with derangement of the bowels; by regulating which its reproduction can only be obviated. All tonsillar concretions are distinguished by a fœtid stercoraceous smell.

*The submaxillary and sublingual glands* are less frequently diseased than the lymphatic glands placed around them. They, however, sometimes inflame, and give rise to a painful swelling under the tongue or jaw: the secretory function of the gland is more or less interrupted. Resolution or induration will commonly be the consequence. It is the opinion of Gariot, a French writer, that the secreting part of a gland cannot suppurate: if so, the matter must be formed by the cellular structure of the organ; and as this constitutes the conducting medium of the blood-vessels, it follows, that general or partial destruction of the gland must take place, according to the extent of the suppuration. In removing the submaxillary gland, the surgeon should recollect, that it is sometimes connected to the sublingual by a communicating slip which must be divided, or the latter also may be needlessly torn away. The calculi which form in these glands, causing a chronic irregular swelling beneath the tongue, may be readily detected, and removed by an incision beside the frenum. A more dangerous disease [Ranula] is induced when the orifice of the sublingual duct becomes obstructed. There is, at first, a small and painful papilla; by the gradual enlargement of which the tongue is, after a time, compressed against the roof of the mouth; and speech, breathing, and deglutition, are impeded: but the tumor presently gives way, and perfect relief is obtained by the discharge of a transparent glairy fluid. This relief, however, is temporary: for the opening again closes; the tumor is reproduced and runs through its former course, but pours out each time, less fluid than before; and the tongue resumes its orginal situation in a proportionately less perfect manner. At length, by frequent repetition of this process, the swelling acquires solidity; increases rapidly; displacing the tongue, turning back its apex so as to incommode breathing and deglutition by pressure upon the epiglottis, and projecting beneath the jaw. In the early stage of the disease, ere thickening of the cyst have taken place, simple treatment will suffice. The sac must be completely laid open, and its internal surface irritated by proper applications, so as to obliterate the cavity and destroy its glandular function. The sore must be healed from the bottom; and, when granulations have arisen, the employment of stimulants must be abandoned. If great indurations, from the advanced state of the case, exist, the tumor should be laid open, and the callous portions removed with the knife or destroyed by actual or potential cautery, so as to induce a granulating sore. When the tumour is seated in the sublingual gland, it protrudes into the mouth; because the mylo-hyoid muscle obstructs its descent toward the throat; but it presents beneath the jaw, when occupying the submaxillary, for that same muscle, under these circumstances, impedes its progress toward the mouth. In the former case, the surgeon would operate on the swelling below the tongue; in the latter, below the jaw.

*Parotid gland.* This organ, when diseased, impedes the motions of the lower jaw: the tumor, from the influence of the fascia, has a flat surface. Mr. Burns asserted that extirpation of this gland is utterly impracticable; and that, in the cases in which this operation is said to have been performed, the tumor removed must have consisted of the conglobate gland, before described as intimately connected with the parotid,* in an enlarged and morbid state. Now, if Mr. Burns meant that every molecule of the glandular substance cannot be cleared away by the operator, the assertion is probably correct; but that the mass of the organ, when unequivocally contaminated by *a malignant affection,* may be so completely removed as to arrest permanently the diffusion of the poison, we have the best possible reason for believing, the evidence of absolute demonstration. But we shall not pause now to discuss the subject. The case to which we allude, with comments on the operation, will, ere long, be communicated by one of our Editors. It is the conglobate gland, imbedded in the centre of the parotid, which when enlarged is so frequently mistaken, according to Mr. Burns' statement, and even extirpated, for a tumor of the salivary organ itself. The other, situated beneath the dependant lobe of the parotid, when diseased, forms a tumor just below, and somewhat behind the angle of the jaw: it is not circumscribed; is immoveable, even at first; but may be easily extirpated, if not very large. It will require to be detached by the fingers from the external carotid, with which it will be connected posteriorly. Disease of the lower lobe of the parotid must be distinguished from this lymphatic tumor. The former sometimes becomes sacculated, lodging a collection of saliva. The swelling begins behind the angle of the jaw, and extends downward and laterally: it is tense, from being covered by the fascia; its fluctuation obscure. This kind of tumor, being simple in its early stage, will only require evacuation of its contents by incision, and irritation of its internal surface by a seton or stimulating injections.

---

* See our former Article, page 61.

A

We must take care not to confound dilatation of the internal jugular vein, which sometimes occurs at this part, with the parotic enlargement. The former may be completely emptied by pressure between the fingers; the latter cannot. In removing a tumor behind the angle of the jaw, the lobe of the parotid should be remembered and avoided; as a wound of it might give rise to salivary fistula. The central conglobate gland of the parotid may swell spontaneously; from mechanical violence; or absorption of irritating matter from the adjacent parts. The tumor, situated between the jaw and mastoid process, is ill defined, and accompanied by a sensation of pain and stiffness. Continuing to enlarge, it produces absorption of the parotid by pressure; and ultimately taking its place, becomes as difficult of extirpation. It is only in the incipient stage, while this tumor remains small and unconnected with its sheath, that, according to Mr. Burns, it can be removed; and, even then, he describes the operation as difficult and dangerous. The subsequent formation of salivary fistula will be obviated by exercising, for a considerable time, steady pressure on the wound of the parotid gland. In the advanced stages of the affection, the tumor becomes blended with its containing sheath; the resistance at first opposed, by the pterygoid muscles and ligament of the jaw, to its encroachments on the larynx and pharynx, yields; and the disease "spreads in every direction." It is the enlargement of this glandular body which, as we have before observed, is often, in Mr. Burns' opinion, confounded with disease of the parotid itself. He has twice seen it affected with fungus hæmatodes. Tumors, implicating the parotid duct, arise principally in two places: either where that tube traverses the masseter muscle; in which case the swelling occupies one of the conglobate glands beside the *socia parotidis:* or where the duct passes from the border of the masseter to the buccinator muscle; and here the tumor is formed by a morbid affection of the mass of adipose substance situated in the hollow between those muscles, or of a lymphatic gland imbedded in it. Upon some tumors of the facial region it would be madness to operate, especially when they are adherent, deeply connected; and induration of the buccal membrane has taken place. With respect to facial tumors, the parotid duct may be situated anteriorly; posteriorly; may be pushed aside; or, where the texture of the morbid mass is soft, be buried with the facial vein in it. The preservation of the duct should always be attended to in the removal of a *simple* swelling; and this object will be facilitated by exposing it between the diseased mass and parotid gland. Its connections with the former are then to be cautiously traced and destroyed: in this part of the operation, the denseness of its structure will direct the surgeon. When the duct is situated exteriorly to the capsule of the tumor, it will be obviously proper to remove the latter entire. The division of the facial vein is a matter of no consequence. If the tumor, involving the duct, be of a *specific* nature, it will be the safest and most politic to sacrifice the latter. All morbid enlargements, situated between the masseter and buccinator muscles, should be extirpated early. No time should be lost in attempts at discuss on, which commonly prove unavailing.

*Operation for hare lip.* It is too commonly supposed that, in passing the pins through the wound, preservation of contiguity of the cut surfaces forms the sole object; but prevention of hæmorrhage is one, little less important. The operator, therefore, instead of introducing the pins in front of the coronary artery, should insinuate them between the vessel and the membrane of the lip. Thus, no gaping wound will be left behind; and bleeding cannot take place. For want of this precaution, Mr. Burns had known the stomach of a patient filled with the blood insidiously escaping into the mouth from the divided artery, after the operation was completed; and the wound torn open by the consequent efforts to vomit. If fine sewing needles are employed, the mark left will be very trifling; and their extraction is facilitated by means of a thread passed through the eye. Hence they are, perhaps, preferable to gold or silver needles.

*Operation for fistula lachrymalis.* In our former article,* we described the mode of introducing an instrument from the nostril into the nasal duct. This operation, under ordinary circumstances, is not very difficult; † but, where obstinate stricture chokes up the canal Mr. Burns recommended that it should be forced by a probe passed through a puncture of the lachrymal sac. The external wound is then to be healed, and the duct kept pervious by the introduction of a curved wire, from the nostril. This procedure resembles in principle the French

---

* See note, page 63.

† Here we cannot agree with Mr. Burns. It has ever appeared to us an arduous and delicate operation.

operation by seton; but is preferable as not "laying the foundation of a fistulous opening into the lachrymal sac." It obviously should be performed early; at least, before the surface of the sac is in a morbid state; or sloughing of the wound may take place, and tedious granulation and a puckered cicatrix be the consequence. Mr. Burns believed that where the obstruction arises from the adhesion induced by *acute* inflammation, it will always be possible to keep the canal open, if it have been rendered pervious at a proper time; but this should never be attempted during the existence of inflammatory action, or after ulceration has become established. In *chronic* thickening of the sac and duct, the operation also will be unavailing. "Here, therefore, it will be preferable to destroy the sac and annihilate the office of the puncta." This state may be recognized by the issue of a clear fluid from the puncta upon pressure: the last drops of which are, at times, turbid, and often milky in a morning. Tumors, forming over the lachrymal sac, may be mistaken for incipient fistula; but they never obstruct the tears, and are distinguished by the facility with which a probe may be passed up the nasal duct. They are commonly sacculated, and contain "a melicerous looking matter," sometimes mixed with hair, or hydatids. It will be necessary to remove the fore part of the cyst with the knife, and destroy the remainder with caustic. The latter part of the operation must not be omitted: for, when the cyst is exposed without removal, great constitutional derangement sometimes ensues in an irritable subject. Consequences equally pernicious may result from allowing the cyst to burst spontaneously. Fungi and induration of the surrounding parts will succeed; and these will require to be destroyed with the knife and caustic. The surgeon must guard against injury of the lachrymal sac from immoderate application of the latter substance.

*The Eye.* Two cases of malignant disease of this organ are detailed by Mr. Burns. The first is fungus hæmatodes; but as it has before appeared in Mr. Wardrop's work, we shall merely observe that extirpation of the eye served only to retard, for a time, the ravages of the malady; and that the antrum, the liver, and parts above the kidnies, presented tumors, the morbid structure of which precisely resembled that of the eye. The optic nerve within the cranium was as thick as the little finger, and as dark in colour as that part of it in the orbit. The junction of the nerves was so much enlarged that it formed a tumor projecting into the third ventricle." Mr. Burns adds, that he "found the dark colour extending much beyond the point where the nerves join, *but confined to the left side or to the nerve of the affected eye.*" The accuracy of this observation we cannot doubt; and how far it tends to decide the long agitated question respecting the decussation of the optic nerves, is sufficiently obvious. The other was an anomalous but terrible affection of the eye: its virulence was only exasperated by an operation. After death, the absorbent glands were found uncontaminated; but the diseased action had extended to the *dura mater*, lining membrane of the nasal sinuses, lachrymal gland on the opposite side, and contents of the orbit from which the morbid eye had been removed during life; in fact, to "parts dissimilar in texture, and, in so far as we know, unconnected by absorbents."

*Arteriotomy.* In opening the temporal artery, Mr. Burns recommended that a quantity of blood should be intercepted by pressure on a portion of the vessel, with the finger and thumb of the left hand properly placed. An incision on the artery, half an inch in length, is next to be made with a scalpel; and its canal opened by a lancet. The bleeding may be arrested by compress and bandage; or by cutting across the vessel, at the *lowest* point of the wound. A touch with oil of turpentine would suffice to check any retnrn of hæmorrhage. Mr. Burns often applied merely adhesive plaster over the wound.

Of the seven plates, destined to elucidate the surgical pathology of the neck and face, the three first are particularly valuable, and entitled to attention; as they display a modification of aortic aneurism which has been, and again may be, confounded with aneurism of the carotid and subclavian arteries. The two figures of the fifth plate are also delineated with uncommon clearness.

Here closes an examination, by far the most laborious, though not the longest, upon which it has ever been our lot to enter. And never did we quit a work with fewer feelings of self complacency and satisfaction, or with more painful consciousness of having traced but a very imperfect outline of the original. The importance and variety of the subjects to be discussed, and the straggling and desultory style of composition, unfortunately adopted by Mr. Burns, opposed obstacles to a *rigorous analysis;* which have, many times, put our patience most severely to the test; and which nothing but a determination to preserve inviolate the solemn pledge given to the public respecting our critical labours, could have excited us to overcome. Mr Burns' book, indeed, may not inaptly be compared to a mine, wherein are contained divers substances, excellent in their kind, but so blended and confused by the sportive hand of nature, as to be greatly deteriorated in beauty and in value. The most costly of these we have laboured to extract, and arrange in the order indicated by their obvious characters, and the intimate relations naturally existing between them. This arrangement might certainly have been rendered more precise; but we were unwilling to destroy the traces of the mode of original *deposition* farther than was necessary to give somewhat of method and perspicuity to our own plan. * Many things have escaped our research, which, on the one hand, too important to be quite overlooked, are, on the other, so minute, or so deeply alloyed, that they will not pay for the expence and trouble of analysis. These we shall leave the reader to seek, and separate, and arrange, for himself

Upon some objectionable parts of the book, which, if the author had been living, would have justified the language of reproof, we shall not comment. Veneration for departed genius is a feeling which we can never sacrifice. Envy may delight in the contemplation of human frailty. Be ours the more grateful task of eulogizing the zeal, and paying homage to the talent, which, had not his thread of existence been prematurely cut short, would have, ere long, raised *Allan Burns* to a distinguished rank in that phalanx of enlightened Scotchmen, to whose exertions Medicine and the tributary Sciences are so deeply indebted; and have rendered his name imperishable!

* The reader on examining the works of Mr. Burns, will find but few traces of the order and arrangement, which we have endeavoured to introduce into this analysis.

